# Supplementary material for: Melanopsin (Opn4) is an oncogene in cutaneous melanoma
Source: Commun Biol. 2022 May 13;5:461. doi: 10.1038/s42003-022-03425-6 (PMC9106662; doi:10.1038/s42003-022-03425-6)
Supplement: Supplementary file 2 — Supplementary Information [file 42003_2022_3425_MOESM2_ESM.pdf]

Supplementary Figures

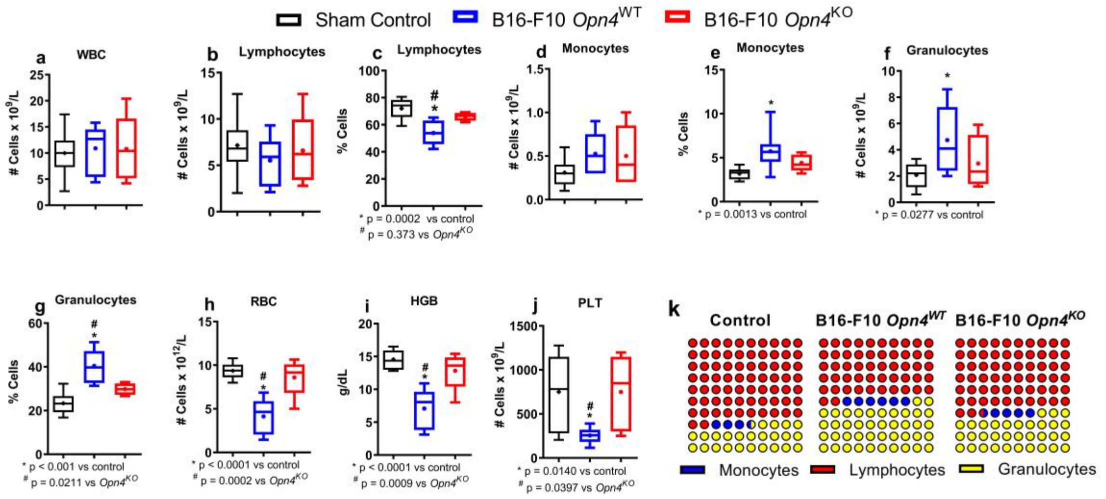

Figure S1. Hemogram analyses comprised of absolute number of white blood cells (a), absolute number and percentage of lymphocytes (b – c), monocytes, (d – e), and granulocytes (f – g) in comparison to total white blood cells. Absolute number of red blood cells (h), hemoglobin content (i), and platelet absolute number (j). A representative graph of monocytes, lymphocytes, and granulocytes is shown in k. In a and b, n = 10, 9, and 5 for sham, B16-F10 *Opn4*^{WT}, and B16-F10 *Opn4*^{KO} groups, respectively. In c, n = 9, 9, and 4, respectively. In d, n = 10, 8, and 5, respectively. In e, n = 10, 10, and 5, respectively. In f, n = 8, 9, and 4, respectively. In g, n = 9, 9, and 4, respectively. In h – i, n = 10,10, and 5, respectively. In j, n = 9, 9, and 5, respectively. In every analyzes, the n number is derived from independent samples. Asterisk represents differences between tumor-bearing and sham (control) animals while hashtag indicates differences between *Opn4*^{WT} and *Opn4*^{KO}. p values are shown in each condition.

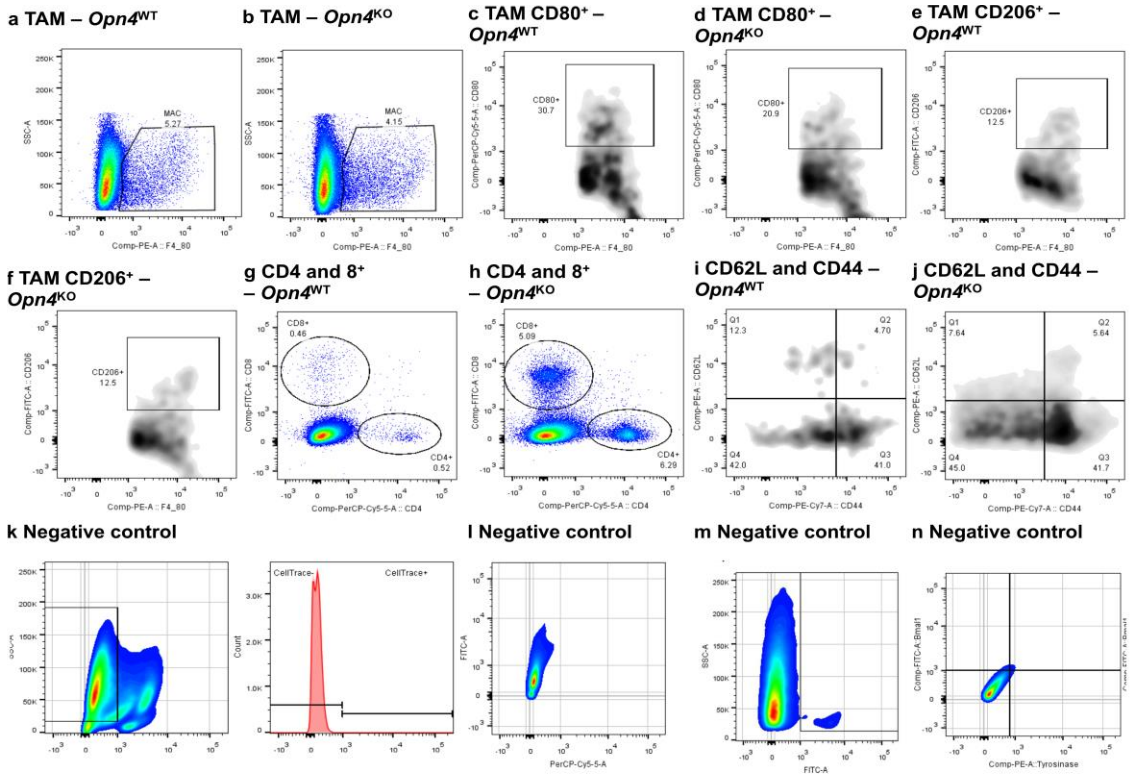

Figure S2. Representative flow cytometry gates of total (a – b), M1 (c – d, CD80+) and M2 (e – f, 206+) macrophages, and CD4+ and CD8+ naïve (g – h), central (CD44+ and CD62L+), and effector memory (CD44+ and CD62L-, i – j) lymphocytes of tumor and spleen samples from *Opn4*^{WT} and *Opn4*^{KO} tumor-bearing mice. k) Negative control of CellTrace experiment (Described in Fig. 2). l) Negative control of cell cycle experiment using BrDU and 7-AAD (Described in Fig. 2). m) Negative control of *in vitro* and n) *in vivo* experiments (Described in Fig. 3 and 5, respectively).

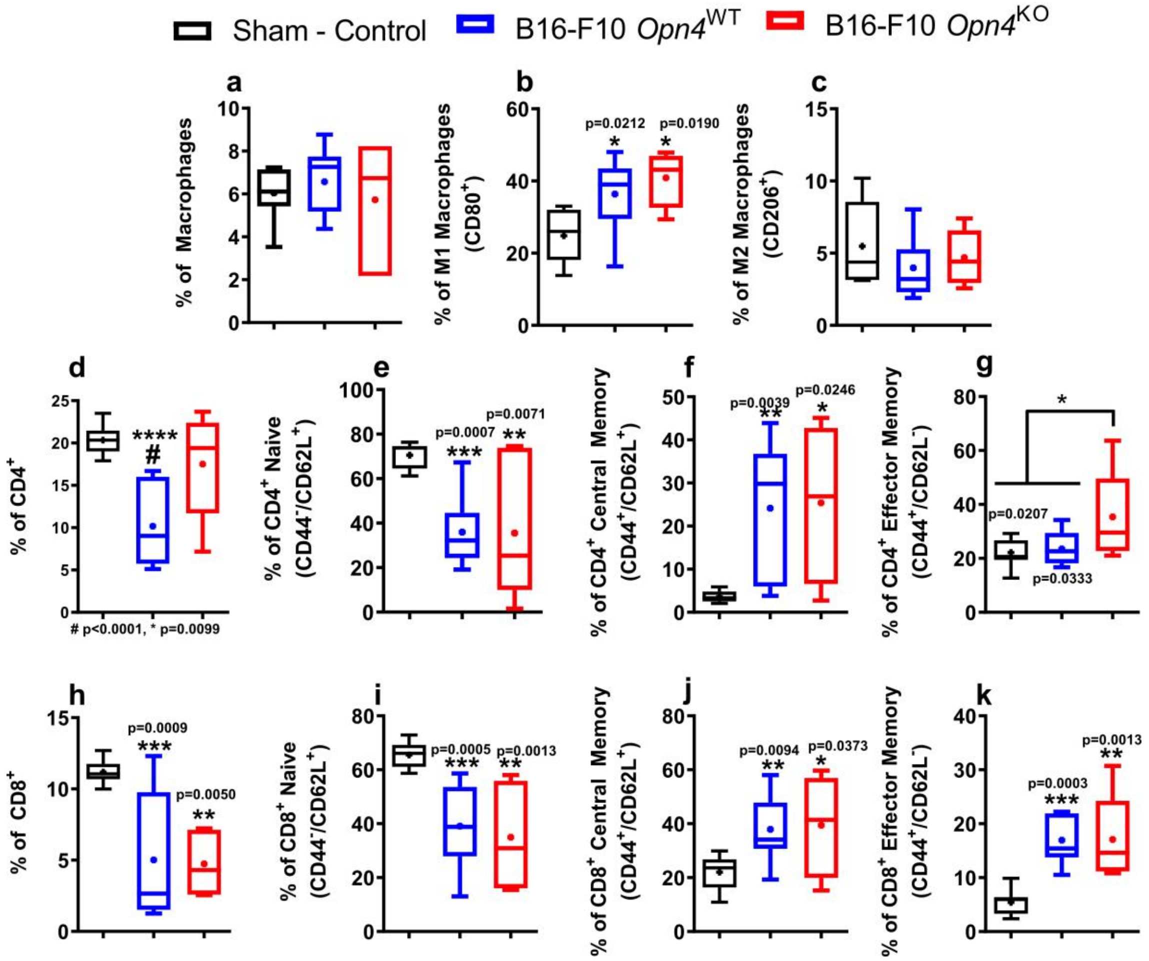

Figure S3. Evaluation of immune system cells in spleen of sham control, *Opn4*^{KO} and *Opn4*^{WT} tumor-bearing mice. Frequency of macrophages (a – c), T CD4+ (d – g), and CD8+ (h – k) lymphocytes and their respective subtypes in spleens of *Opn4*^{KO} and *Opn4*^{WT} tumor-bearing and sham control mice. Subtypes of each cell population are indicated in the Y axis. In a, n = 8, 13, and 7 for sham, B16-F10 *Opn4*^{WT}, and B16-F10 *Opn4*^{KO} groups, respectively. In b, n = 9, 12, and 4, respectively. In c, n = 8, 11, and 6, respectively. In d, n = 10, 12, and 5, respectively. In e, n = 9, 11, and 6, respectively. In f, n = 9, 12, and 4, respectively. In g, n = 10, 12, and 6, respectively. In h, n = 8, 12, and 5, respectively. In i, n = 9, 12, and 5, respectively. In j, n = 10, 11, and 4, respectively. In k, n = 8, 9, and 5, respectively. In every analyzes, the n number is derived from independent samples. Representative gate strategy is shown in Figure S2. Asterisks represent differences between *Opn4*^{WT} and *Opn4*^{KO} tumor-bearing mice compared to sham control mice. Hashtag represents differences between *Opn4*^{WT} tumor-bearing mice and sham control animals. Brackets indicate the differences between *Opn4*^{KO} tumor-bearing mice and the remaining groups.

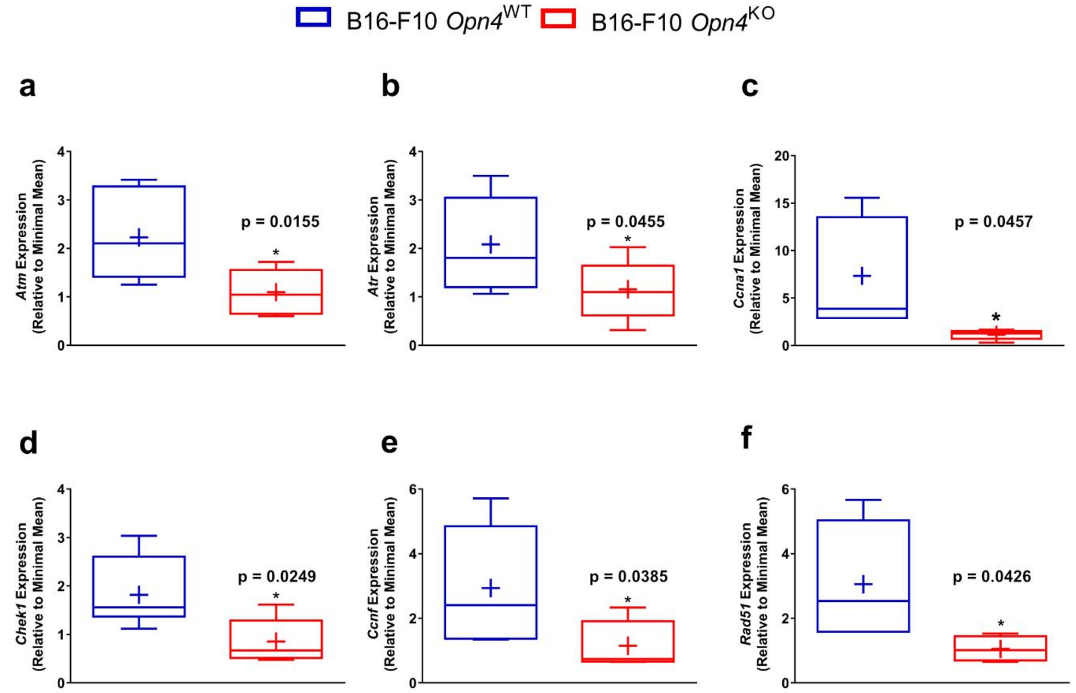

Figure S4. Gene expression in tumor bulk of *Opn4*^{KO} and *Opn4*^{WT} tumors**.** a – f) *In vivo* expression of cell cycle-related genes of *Opn4*^{WT} and *Opn4*^{KO} tumor bulk. In a, n = 7 and 6 for *Opn4*^{KO} and *Opn4*^{WT} tumors, respectively; in b, n = 8 and 7, respectively. in c, n = 5 for both groups; in d, n = 7 and 5, respectively. in e, n = 8 and 6, respectively. in f, n = 7 and 5, respectively. In every analyzes, the n number is derived from independent sample. Gene name is shown in the Y-axis. * $p < 0.05$.

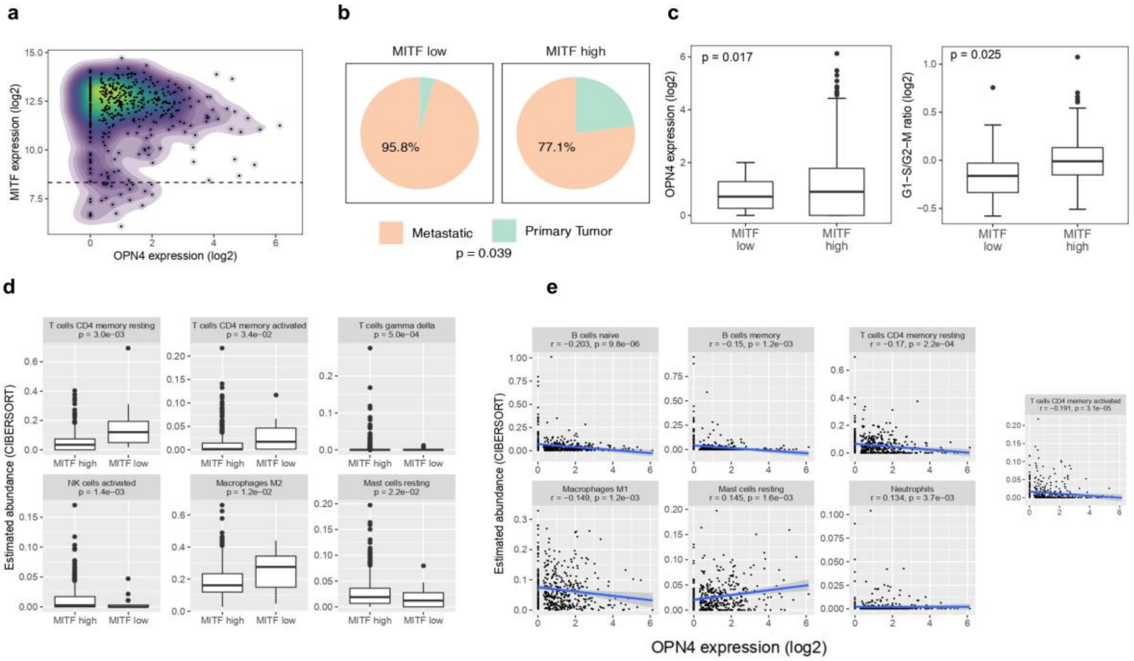

Figure S5. Bioinformatics analyses of TCGA RNA-seq data from melanoma tumors. a) *MITF* and *OPN4* expression in 103 primary melanomas and 368 metastatic melanomas. Tumors were stratified into high and low *MITF* expression based on the 5th percentil (dashed line). b) Proportion of *MITF* high and low tumors in primary and metastatic disease. c) *OPN4* expression and the inferred G1/S vs. G2/M ratio in low and high *MITF* tumors. d) Abundance of tumor-infiltrating immune cells estimated by the CIBERSORT algorithm in high and low *MITF* tumors. e) Correlation analyses between estimated abundances and *OPN4* expression.

Supplementary Table S1

Drug's Name	Target	Vehicle	Concentration used
BAPTA-AM	Calcium chelator	DMSO	10 µM
Dexamethasone	Specific GR activator	PBS	200 nM
Forskolin	Adenylyl cyclase activator	DMSO	10 µM
KN-93	Calcium/calmodulin-dependent protein kinase activator	DMSO	9 µM
L-Name	Oxide nitric synthase inhibitor	PBS	10 – 20 mM
ODQ -	Guanylyl cyclase inhibitor	DMSO	50 µM
U-73122	Phospholipase C inhibitor	DMSO	10 µM

DMSO concentration was never higher than 2% during experiments. Drugs' concentration was based on a previous study [44].

Supplementary Table S2

Templates (Access numbers)	Primers and probes (5' – 3')
Atm (NM_007499.3)	For: AACCATGCTTGCTGTTGTCG<br>Rev: AATCCAGCCAGAAAGCGTCA
Atr (NM_019864.1)	For: CCTCAAACCGCTTTTTCGCA<br>Rev: ATCCGGCCTTTTGTTGAGACT
Bmal1 (NM_001243048)	For: AAGCTTCTGCACAATCCACAGCAC<br>Rev: TGTCTGGCTCATTGTCTTCGTCCA<br>Probe: 5'-/5HEX/AAAGCTGGCCACCCACGAAGATGGG/BHQ_1/-3'
Ccna1 (NM_007628.3)	For: GAAATTGCAGCTTGTCGGGA<br>Rev: TGCCAGGACTTTGAGTAGCAG
Ccnf (NM_007634.4)	For: TCCACGATGATGCACCCAAA<br>Rev: TTTCTCGCTTCCGTTTGCTC
Chek1 (NM_007691.5)	For: TGTGCATTTGGATTCCTGTGG<br>Rev: CTATGGCCCGCTTCATGTCTA
Gzmf NM_010374	For: GCTGGGGGAGAACATCCATC<br>Rev: TGTCCTGTTTAGCCCATAGGT
Il-10 NM_010548	For: GCTCTTACTGACTGGCATGAG<br>Rev: CGCAGCTCTAGGAGCATGTG
Il-1β NM_008361	For: GCAACTGTTCCTGAACTCAACT<br>Rev: ATCTTTTGGGGTCCGTCAACT
Il-6 NM_010559	For: CCTGAGACTCAAGCAGAAATGG<br>Rev: AGAAGGAAGGTCGGCTTCAGT
Mitf (NM_001113198.1)	For: CCCAGGTATGAACACGCACT<br>Rev: CTGTGGGGAAAATACACGCTG
Prf1 NM_011073	For: AGCACAAGTTCGTGCCAGG<br>Rev: GCGTCTCTCATTAGGGAGTTTTT
Rad51 (NM_011234.5)	For: GCTGTTGCTTATGCACCGAA<br>Rev: AACTCAGTTGCCGTGGTGAA
Rpl37a (NM_009084.4)	For: GCATGAAAACAGTGGCCGGT<br>Rev: CAGGGTCACACAGTATGTCTCAAAA
Tgf-β NM_009367	For: CTTCGACGTGACAGACGCT<br>Rev: GCAGGGGCAGTGTAAACTTATT

All primers were used at 300 nM except for the *Bmal1* probe (200 nM)
