# Supplementary material for: Melanopsin (Opn4) is an oncogene in cutaneous melanoma
Source: Commun Biol. 2022 May 13;5:461. doi: 10.1038/s42003-022-03425-6 (PMC9106662; doi:10.1038/s42003-022-03425-6)
Supplement: Supplementary file 3 — Description of Additional Supplementary Files [file 42003_2022_3425_MOESM3_ESM.pdf]

Description of Additional Supplementary Files

File name: Supplementary Data 1
Description: $Opn4^{WT}$ tumor proteome based on proteins identified in at least two of four biological replicates.
File name: Supplementary Data 2
Description: $Opn4^{KO}$ tumor proteome based on proteins identified in at least two of four biological replicates.
File name: Supplementary Data 3
Description: Label-free quantification (LFQ) analysis of 992 identified proteins shared among the groups $Opn4^{WT}$ and $Opn4^{KO}$.
File name: Supplementary Data 4
Description: Information (Uniprot Entry, Gene, Regulation, Average Area - Avg. Area, and notes) of differentially expressed or quantified proteins related to circadian rhythm, cell cycle (GO-term biological process), and MITF regulation, according to KEGG database, between $Opn4^{WT}$ and $Opn4^{KO}$ tumors.
File name: Supplementary Data 5
Description: Experimental data used to create the graphs from Figure 1 to 5 and from S1 to S5.
