# Supplementary material for: Melanopsin (Opn4) is an oncogene in cutaneous melanoma
Source: Commun Biol. 2022 May 13;5:461. doi: 10.1038/s42003-022-03425-6 (PMC9106662; doi:10.1038/s42003-022-03425-6)
Supplement: Supplementary file 6 — Reporting summary [file 42003_2022_3425_MOESM6_ESM.pdf]

nature portfolio

Corresponding author(s): de Assis, LVM

Last updated by author(s): Mar 27, 2022

Reporting Summary

Nature Portfolio wishes to improve the reproducibility of the work that we publish. This form provides structure for consistency and transparency in reporting. For further information on Nature Portfolio policies, see our Editorial Policies and the Editorial Policy Checklist.

Statistics

For all statistical analyses, confirm that the following items are present in the figure legend, table legend, main text, or Methods section.

n/a	Confirmed	
☐	☒	The exact sample size (*n*) for each experimental group/condition, given as a discrete number and unit of measurement
☐	☒	A statement on whether measurements were taken from distinct samples or whether the same sample was measured repeatedly
☐	☒	The statistical test(s) used AND whether they are one- or two-sided<br>*Only common tests should be described solely by name; describe more complex techniques in the Methods section.*
☐	☒	A description of all covariates tested
☐	☒	A description of any assumptions or corrections, such as tests of normality and adjustment for multiple comparisons
☐	☒	A full description of the statistical parameters including central tendency (e.g. means) or other basic estimates (e.g. regression coefficient) AND variation (e.g. standard deviation) or associated estimates of uncertainty (e.g. confidence intervals)
☐	☒	For null hypothesis testing, the test statistic (e.g. *F*, *t*, *r*) with confidence intervals, effect sizes, degrees of freedom and *P* value noted<br>*Give P values as exact values whenever suitable.*
☒	☐	For Bayesian analysis, information on the choice of priors and Markov chain Monte Carlo settings
☒	☐	For hierarchical and complex designs, identification of the appropriate level for tests and full reporting of outcomes
☒	☐	Estimates of effect sizes (e.g. Cohen's *d*, Pearson's *r*), indicating how they were calculated

Our web collection on statistics for biologists contains articles on many of the points above.

Software and code

Policy information about availability of computer code

Data collection	Raw data was obtained directly from the equipaments and processed in excel
Data analysis	Data was analyzed in GraphPad Prism version 7.0

For manuscripts utilizing custom algorithms or software that are central to the research but not yet described in published literature, software must be made available to editors and reviewers. We strongly encourage code deposition in a community repository (e.g. GitHub). See the Nature Portfolio guidelines for submitting code & software for further information.

Data

Policy information about availability of data

All manuscripts must include a data availability statement. This statement should provide the following information, where applicable:

- Accession codes, unique identifiers, or web links for publicly available datasets
- A description of any restrictions on data availability
- For clinical datasets or third party data, please ensure that the statement adheres to our policy

The raw MS data associated with this manuscript have been submitted to a public repository (the Mass Spectrometry Interactive Virtual Environment – MassIVE, http://massive.ucsd.edu) and deposited to the ProteomeXchange Consortium (http://www.proteomexchange.org/). These data are associated with the identifier MassIVE ID MSV000088579 and Proteome Exchange ID PXD030477.

Field-specific reporting

Please select the one below that is the best fit for your research. If you are not sure, read the appropriate sections before making your selection.

☒ Life sciences ☐ Behavioural & social sciences ☐ Ecological, evolutionary & environmental sciences

For a reference copy of the document with all sections, see nature.com/documents/nr-reporting-summary-flat.pdf

Life sciences study design

All studies must disclose on these points even when the disclosure is negative.

Sample size	Optimal sample size was empirically determined to be at least 6 samples for in vitro studies. For in vivo studies, due to variation in tumor development, optimal sample size was determined to be at least 7 samples.
Data exclusions	In all analyzes, values were excluded when higher or lower than mean ± 2 standard deviation (SD).
Replication	All experiments were repeated at least twice
Randomization	It was not performed
Blinding	It was not performed

Reporting for specific materials, systems and methods

We require information from authors about some types of materials, experimental systems and methods used in many studies. Here, indicate whether each material, system or method listed is relevant to your study. If you are not sure if a list item applies to your research, read the appropriate section before selecting a response.

Materials & experimental systems

n/a	Involved in the study	
☐	☒	Antibodies
☐	☒	Eukaryotic cell lines
☒	☐	Palaeontology and archaeology
☐	☒	Animals and other organisms
☒	☐	Human research participants
☒	☐	Clinical data
☒	☐	Dual use research of concern

Methods

n/a	Involved in the study	
☒	☐	ChIP-seq
☐	☒	Flow cytometry
☒	☐	MRI-based neuroimaging

Antibodies

Antibodies used	anti-BrdU antibody provided in kit 559619 from BD Company;<br>For flow cytometry all from Biolegend: APC-Cy7 anti-Mouse F4/80 (Cat no. 123118), PerCP-Cy5.5 anti-Mouse CD80 (Cat no. 194722), FITC anti-mouse CD206 (Cat no.141704), FITC anti-mouse CD4 (Cat no. 100509), PE-Cy7 anti-mouse CD44 (Cat no. 560569), PE anti-mouse CD62L, (Cat no. RM4304), and APC anti-mouse CD8 (Cat no. MCD0805, Invitrogen, USA).<br>For tyrosinase (goat polyclonal, Santa Cruz, USA SC 18182), BMAL1 (1:100, rabbit polyclonal, ABCAM, ab93806). Secondary antibodies anti-goat (555 nm) and anti-rabbit (488 nm) (both Alexa Fluor ThermoFisher, USA)
Validation	All antibodies were used according to the manufacturer's instruction followed by serial dilution and cytometry analyzes to determine the best concentration

Eukaryotic cell lines

Policy information about cell lines

Cell line source(s)	B16-F10 cells were originally donated by Prof Chammas from University of Sao Paulo (USP)
Authentication	No authentication was performed
Mycoplasma contamination	Cells were not tested for mycoplasma contamination
Commonly misidentified lines (See ICLAC register)	NA

Animals and other organisms

Policy information about studies involving animals; ARRIVE guidelines recommended for reporting animal research

Laboratory animals	3 to 8 months-old C57BL/6J
Wild animals	NA
Field-collected samples	NA
Ethics oversight	NA

Note that full information on the approval of the study protocol must also be provided in the manuscript.

Flow Cytometry

Plots

Confirm that:

☒ The axis labels state the marker and fluorochrome used (e.g. CD4-FITC).

☒ The axis scales are clearly visible. Include numbers along axes only for bottom left plot of group (a 'group' is an analysis of identical markers).

☒ All plots are contour plots with outliers or pseudocolor plots.

☒ A numerical value for number of cells or percentage (with statistics) is provided.

Methodology

Sample preparation	For in vitro experiments, after experimental manipulation (see M&M), all cells were fixed in cytoperm/fix solution. For in vivo experiments, freshly collected cells were prepared, as described in the manuscript. All cells were then loaded in to the equipment and data was obtained.
Instrument	Canto II flow cytometer (BD Biosciences, USA)
Software	DiVA 8 acquisition software.
Cell population abundance	No cell sorting was performed
Gating strategy	Cells were gated using FSC and SCC, duplets were excluded using FSC-H vs FSC-A. Non-viabible cells were also excluded using Live/Dead dye (Fixable aqua 405 nm, Invitrogen, USA)

☒ Tick this box to confirm that a figure exemplifying the gating strategy is provided in the Supplementary Information.
